# The Careful Treatment of Stumps

**Published:** 1918-10

**Authors:** 


***The Careful Treatment of Stumps.*** By P. DESFOSSES. Trans. and Abs. from *La Presse Médicale*, June 10, 1918.

The author insists that the orthopedic care of a stump should be continuous from the time of amputation until the patient enters the re-educational school.

During the period of healing, the stump should be so placed that in case ankylosis occurs it may still be utilized. To prevent ankylosis the position of the stump should be frequently changed during the operation, and later the stump should often be exercised.

The healing should not be left to itself, but the surgeon should do everything possible to bring the edges of the wound together. The method employed to this end varies with the needs of the case.

If the healing process is going well, the edges may be brought together with bands of adhesive plaster.

If the flaps are too far apart, each flap may be provided with rows of hooks. Elastic lacings are then applied to draw the edges together.

If there is recession of the soft parts, and especially of the skin, an adhesive bracelet supplied with hooks or some means of attach ing an extension apparatus is applied, and the skin is thus held by a steady tension throughout the healing period.

It is a mistake to keep the stump in repose during healing, or to confine walking to crutches. On the contrary, the stump should be utilized at the earliest opportunity, as it is to be used permanently. For this purpose temporary means of prothesis are devised, so adjusted as to relieve the wound of all strain. These appliances, suspended from the shoulder by straps, enable the patient to practise walking with the help of only a cane.

The author reviews the many accidents which may prevent healing, especially suppuration, due to foreign bodies, sequestra, or osteomyelitis, both terminal and lateral. Such conditions present much difficulty in operation. The stump development may also be complicated by abnormal but healthy bone growth, which may not be troublesome. It may often be neglected entirely.

Even if healing has been completely successful, the stump may still be useless. Painful stumps may be caused by too much soft tissues at the end of the bone. If everything appears normal, the pain must be attributed to nerve ends near the surface. If the pain is felt in the scar itself, Tuffier considers resection of the scar sufficient to reveal the nerve disorder. In case there is a general sensitiveness of the stump, Tuffier practises a full flap incision at the level of the maximum sensitiveness, with resection of the nerve.

When the healing has been completed in perfect condition, considerable care is still necessary before the stump is ready for an artificial limb, especially if of the American type. The skin must be rendered supple by bathing, massage and ointments. Massage and electrical treatment help to impart tone to the muscles, and simple appliances may be devised to remedy stiffness of the joints and mild cases of ankylosis. These must be supplemented by massage and by passive and active movements.

The author describes with much detail the temporary appliances used at La Panne by the Belgian surgeons to secure the necessary compression of the soft parts, and to start the reduction of the limb. These may be adjusted to follow the evolution of the stump, and so act as a supplement to surgical treatment as well as a means of early reduction. They are roughly and cheaply constructed with plastered bands, iron hinges, metal setting, wooden crutches, and leather straps and bands.

